# Crystal structure of a Co^II^ coordination polymer: *catena*-poly[[μ-aqua-bis­(μ-2-methyl­propano­ato)-κ^2^ *O*:*O*′;κ^2^ *O*:*O*-cobalt(II)] monohydrate]

**DOI:** 10.1107/S2056989017001360

**Published:** 2017-02-03

**Authors:** Andrei I. Fischer, Vladislav V. Gurzhiy, Julia V. Aleksandrova, Maria I. Pakina

**Affiliations:** a St Petersburg State Institute of Technology, Moskovsky pr. 26, 190013, St Petersburg, Russian Federation; b Institute of Earth Sciences, St Petersburg State University, University Emb. 7/9, 199034, St Petersburg, Russian Federation

**Keywords:** crystal structure, polynuclear complexes, coordination polymer, cobalt carboxyl­ates, cobalt(II) isobutyrate dihydrate

## Abstract

In the title cobalt(II) coordination polymer with isobutyrate ligands, {[Co{CH(CH_3_)_2_CO_2_}_2_(H_2_O)]·H_2_O}_*n*_, the Co^2+^ ion is hexa­coordinated in a slightly distorted octa­hedral coordination environment defined by two O atoms from two bridging water mol­ecules and four O atoms from four bridging carboxyl­ate ligands. The carboxyl­ates adopt two different coordination modes, μ-(κ^2^ *O*:*O*′) and μ-(κ^2^ *O*:*O*), forming a one-dimensional polymeric chain extending along [010]. The intra-chain cobalt⋯cobalt separation is 3.2029 (2) Å. The polymeric chains are linked by hydrogen bonds involving the water mol­ecules of solvation, giving a two-dimensional network structure lying parallel to (100).

## Abstract 2

The present paper describes the synthesis and crystal structure of a cobalt(II) isobutyrate dihydrate, based on a slightly distorted CoO_6_ repeat unit comprising four bridging carboxyl­ate O-atom donors and two bridging water donors, giving one-dimensional polymeric chains with composition {[Co{(CH_3_)_2_CHCO_2_}_2_(H_2_O)]·H_2_O}_*n*_. Hydrogen bonding through the water mol­ecules gives two-dimensional sheets lying parallel to (100).

## Chemical context   

Carboxyl­ate anions still remain a popular choice as bridging ligands because of their ability to form diverse oligo- and polynuclear structures. Oligo- and polynuclear cobalt carboxyl­ates in turn have attracted great attention because of their utilization in homogeneous oxidation catalysis (Gates, 1992 ▸; Parshall & Ittel, 1992 ▸; Partenheimer, 1995 ▸; Ward *et al.*, 2013*a* ▸), and their inter­esting magnetic properties (Ward *et al.*, 2013*b* ▸; Eremenko *et al.*, 2009 ▸). Recently, we have reported on the crystal structures of the hydrated polymeric cobalt(II) propionate (Fischer *et al.*, 2010 ▸) and butyrate (Fischer *et al.*, 2011 ▸), which were prepared by the reaction of cobalt(II) carbonate hydrate with the corresponding aqueous carb­oxy­lic acid. The aim of these studies was to investigate the influence of the steric features of the carboxyl­ate anion on the structure of the resulting compounds. Cobalt(II) carboxyl­ates are of inter­est for our group as starting materials for the synthesis of mixed-valence cobalt carboxyl­ates (Fischer, Kuznetsov & Belyaev, 2012 ▸; Fischer, Kuznetsov, Shchukarev & Belyaev, 2012 ▸). In addition, we intend to examine the catalytic activity of the cobalt(II) carboxyl­ates obtained, which will be used for introduction into the sodalite cages of synthetic NaY zeolites, modified by deca­tionation and dealuminizing methods.

As a part of our ongoing studies on these compounds, we describe here synthesis and crystal structure of the title compound, {[Co{CH(CH_3_)_2_CO_2_}_2_(H_2_O)]·H_2_O}_*n*_, (I).

## Structural commentary   

**Figure 1:**
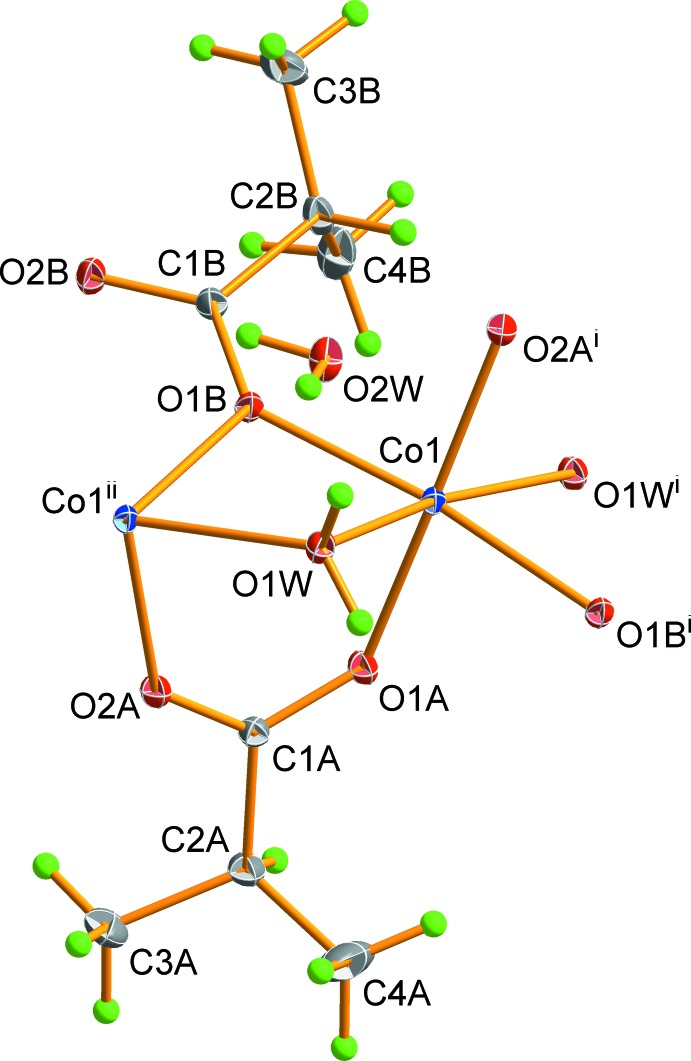
The coordination mode and atom-numbering scheme for (I). Displacement ellipsoids of the non H-atoms are drawn at the 50% probability level, with H atoms shown as spheres of arbitrary radius. [Symmetry codes: (i) −*x* + 1, *y* + 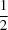, −*z* + 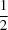; (ii) −*x* + 1, *y* − 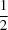, −*z* + 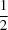.

The structure of (I) contains one independent Co^2+^ cation coordinated by four O atoms from four bridging isobutyrate ligands and two O atoms from two bridging water mol­ecules (O1*W*) in a distorted octa­hedral coordination. A water mol­ecule of solvation (O2*W*) is also present (Fig. 1 ▸). The Co—O bond lengths are in the range 2.0142 (6)–2.1777 (6) Å (Table 1 ▸) and the *cis-*angles about the Co^2+^ atom vary in the range 78.99 (3)–110.31 (2)°. This data correlates with the angles and the distances in cobalt(II) acetate dihydrate which has a similar structure (Jiao *et al.*, 2000 ▸), as well as with the closely related cobalt(II) propionate dihydrate (Fischer *et al.*, 2010 ▸) and cobalt(II) butyrate 1.7-hydrate (Fischer *et al.*, 2011 ▸).
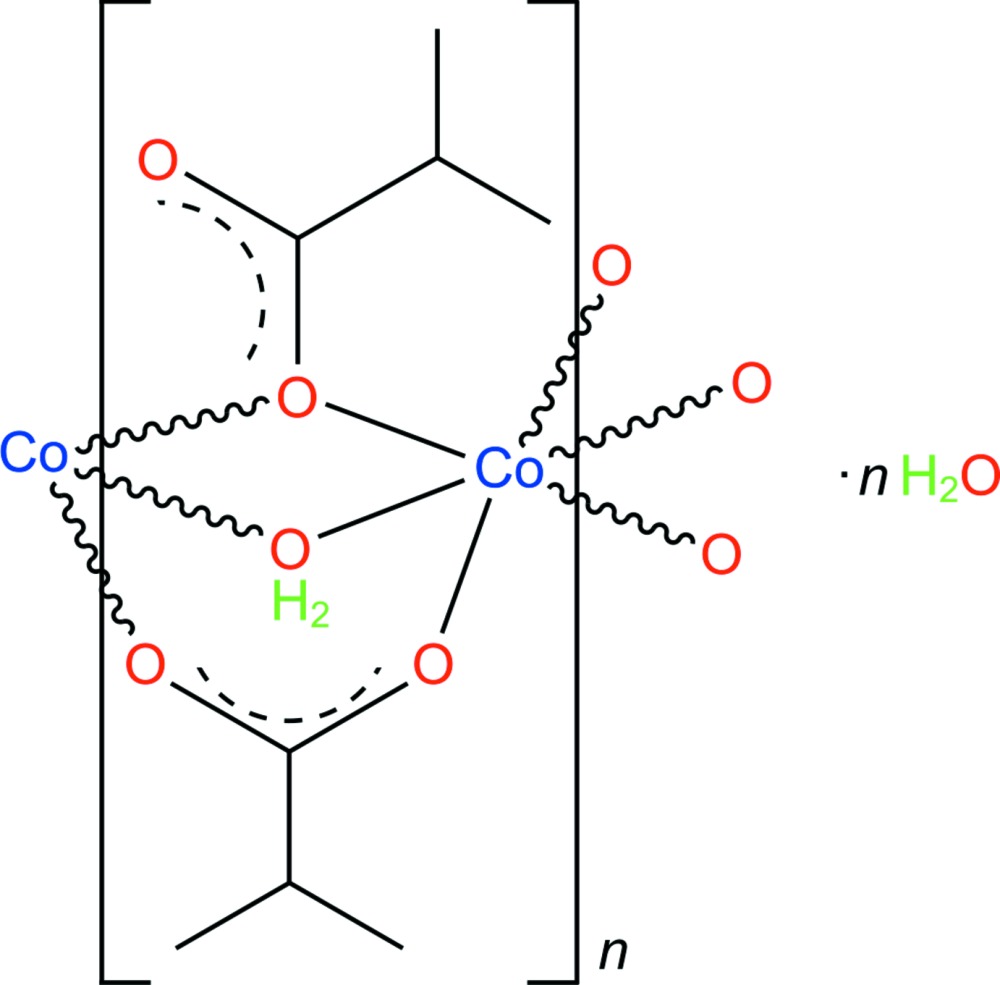


**Figure 2:**
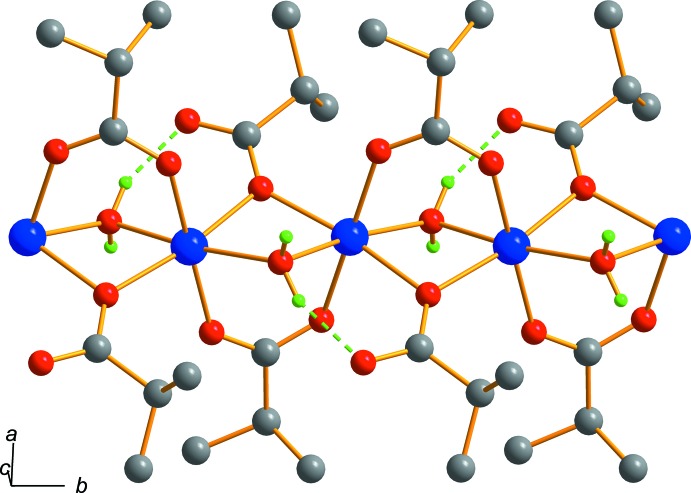
The one-dimensional polymeric structure of (I) extending along [010], with the intra­molecular hydrogen bond shown as a dashed line. The carbon-bound H atoms and the water mol­ecule of solvation have been omitted.

The structure of (I) is based on infinite chains with _∞_[Co(H_2_O)((CH_3_)_2_CHCOO)_2_] composition, extending along [010] (Fig. 2 ▸). The Co⋯Co distance within the chain is 3.2029 (2) Å. The formation of polymeric chains may be a plausible reason for the crystal growth being predominantly along the *b* axis. The bridging carboxyl­ate groups adopt two coordination modes, μ-(κ^2^ *O*:*O*′) and μ-(κ^2^ *O*:*O*). The C—O bond lengths of the first group (involving O1*A* and O2*A*) have close values [1.2755 (10) and 1.2533 (10) Å], whereas those of the second group (involving O1*B* and O2*B*) have a more striking difference [1.2878 (9) and 1.2510 (11) Å]. The carboxyl­ate O2*B* atom of the second group forms an inter-unit hydrogen bond with the bridging water mol­ecule [O1*W*—H⋯O2*B* ^i^ = 2.6206 (9) Å] (Fig. 2 ▸, Table 2 ▸).

## Supra­molecular features   

**Figure 3:**
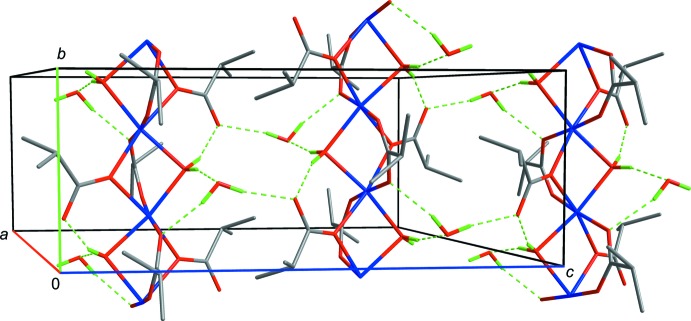
The packing diagram of (I), showing the inter­actions between the coordination polymer chains. Hydrogen bonds are shown as dashed lines. The carbon-bound H atoms are omitted for clarity.

Metal–organic chain polymers are linked together through the water mol­ecule of solvation (O2*W*) by a system of hydrogen bonds, forming a sheet structure arranged parallel to (100) (Table 2 ▸, Fig. 3 ▸). Only weak van der Waals inter­actions link neighboring sheets in the crystal structure.

## Database survey   

A survey of the Cambridge Structural Database (Groom *et al.*, 2016 ▸) reveals only the following related one-dimensional polymeric structures of cobalt(II) carboxyl­ates with composition _∞_[Co(*R*COO)_2_(H_2_O)]: acetate (Jiao *et al.*, 2000 ▸), propionate (Fischer *et al.*, 2010 ▸) and butyrate (Fischer *et al.*, 2011 ▸).

## Synthesis and crystallization   

The title compound was synthesized using a similar procedure as for the synthesis of the analogous carboxyl­ates cobalt(II) propionate dihydrate (Fischer *et al.*, 2010 ▸) and cobalt(II) butyrate 1.7-hydrate (Fischer *et al.*, 2011 ▸). To a mixture of isobutyric acid (8.8 g, 100 mmol) and water (100 ml), an excess of fresh cobalt(II) carbonate hexa­hydrate, CoCO_3_·6H_2_O, (13.6 g, 60 mmol) was added. The reaction mixture was period­ically stirred in an ultrasonic bath at room temperature until the liberation of carbon dioxide ceased. The unreacted CoCO_3_·6H_2_O was removed by filtration, and the filtrate was allowed to stand at room temperature for slow evaporation. Red single crystals of (I) suitable for X-ray diffraction were obtained after several days. The yield was 81%.

## Refinement   

Crystal data, data collection and structure refinement details are summarized in Table 3 ▸. The hydrogen atoms of the water mol­ecules were located from differenc maps and refined in an isotropic approximation with *U*_iso_(H) set to 1.5*U*_eq_(O). Other hydrogen atoms were placed in calculated positions and refined using a riding model with *d*(C—H) = 0.98 Å, *U*_iso_(H) = 1.2*U*_eq_(C) for the tertiary carbon atoms and *d*(C—H) = 0.96 Å, *U*_iso_(H) = 1.5*U*_eq_(C) for the methyl groups.

## Supplementary Material

Crystal structure: contains datablock(s) global, I. DOI: 10.1107/S2056989017001360/zs2371sup1.cif

CCDC reference: 1529830

Additional supporting information:  crystallographic information; 3D view; checkCIF report

## Figures and Tables

**Table 1:** Selected geometric parameters (Å, °)

Co1—O1*A*	2.0449 (6)	Co1—O1*B* ^i^	2.1198 (6)
Co1—O2*A* ^i^	2.0142 (6)	Co1—O1*W*	2.1768 (6)
Co1—O1*B*	2.1100 (6)	Co1—O1*W* ^i^	2.1777 (6)
			
O1*A*—Co1—O1*B*	88.13 (3)	O2*A* ^i^—Co1—O1*W* ^i^	88.33 (3)
O1*A*—Co1—O1*B* ^i^	89.41 (3)	O1*B*—Co1—O1*B* ^i^	170.29 (2)
O1*A*—Co1—O1*W*	92.18 (3)	O1*B*—Co1—O1*W*	79.22 (2)
O1*A*—Co1—O1*W* ^i^	88.29 (3)	O1*B* ^i^—Co1—O1*W*	91.49 (2)
O2*A* ^i^—Co1—O1*A*	175.30 (3)	O1*B*—Co1—O1*W* ^i^	110.31 (2)
O2*A* ^i^—Co1—O1*B*	89.99 (3)	O1*B* ^i^—Co1—O1*W* ^i^	78.99 (2)
O2*A* ^i^—Co1—O1*B* ^i^	93.14 (3)	O1*W*—Co1—O1*W* ^i^	170.46 (2)
O2*A* ^i^—Co1—O1*W*	91.70 (3)		

Symmetry code: (i) 
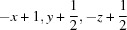
.

**Table 2:** Hydrogen-bond geometry (Å, °)

*D*—H⋯*A*	*D*—H	H⋯*A*	*D*⋯*A*	*D*—H⋯*A*
O1*W*—H1*W*1⋯O2*W*	0.79 (2)	1.91 (2)	2.6638 (10)	161 (2)
O1*W*—H1*W*2⋯O2*B* ^i^	0.88 (2)	1.79 (2)	2.6206 (9)	158 (2)
O2*W*—H2*W*1⋯O1*A* ^ii^	0.86 (1)	2.01 (1)	2.7967 (9)	151 (1)
O2*W*—H2*W*2⋯O2*B* ^iii^	0.88 (1)	1.95 (1)	2.8087 (9)	163 (1)
C2*B*—H2*B*⋯O2*A* ^i^	0.98	2.47	3.3094 (11)	144

Symmetry codes: (i) 
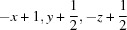
; (ii) 
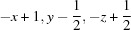
; (iii) 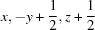.

**Table 3:** Experimental details

Crystal data	
Chemical formula	[Co(C_4_H_7_O_2_)_2_(H_2_O)]·H_2_O
*M*_r_	269.15
Crystal system, space group	Monoclinic, *P*2_1_/*c*
Temperature (K)	100
*a*, *b*, *c* (Å)	11.9999 (4), 6.3815 (2), 16.1374 (6)
β (°)	109.540 (2)
*V* (Å^3^)	1164.59 (7)
*Z*	4
Radiation type	Mo *K*α
μ (mm^−1^)	1.48
Crystal size (mm)	0.35 × 0.15 × 0.1

Data collection	
Diffractometer	Bruker APEXII CCD
Absorption correction	Multi-scan (*SADABS*; Bruker, 2014 ▸)
*T*_min_, *T*_max_	0.304, 0.417
No. of measured, independent and observed [*I* > 2σ(*I*)] reflections	25308, 5082, 4459
*R*_int_	0.070
(sin θ/λ)_max_ (Å^−1^)	0.807

Refinement	
*R*[*F* ^2^ > 2σ(*F* ^2^)], *wR*(*F* ^2^), *S*	0.026, 0.068, 1.03
No. of reflections	5082
No. of parameters	152
No. of restraints	4
H-atom treatment	H atoms treated by a mixture of independent and constrained refinement
Δρ_max_, Δρ_min_ (e Å^−3^)	1.25, −0.51

Computer programs: *APEX2* and *SAINT* (Bruker, 2014 ▸), *SHELXS97* (Sheldrick, 2008 ▸), *SHELXL2012* (Sheldrick, 2015 ▸), *DIAMOND* (Brandenburg, 2012 ▸) and *OLEX2* (Dolomanov *et al.*, 2009 ▸).

## supplementary crystallographic information

### Crystal data

**Table:**

[Co(C_4_H_7_O_2_)_2_(H_2_O)]·H_2_O	*F*(000) = 564
*M**_r_* = 269.15	*D*_x_ = 1.535 Mg m^−^^3^
Monoclinic, *P*2_1_/*c*	Mo *K*α radiation, λ = 0.71073 Å
*a* = 11.9999 (4) Å	Cell parameters from 9959 reflections
*b* = 6.3815 (2) Å	θ = 3.5–49.6°
*c* = 16.1374 (6) Å	µ = 1.48 mm^−^^1^
β = 109.540 (2)°	*T* = 100 K
*V* = 1164.59 (7) Å^3^	Prism, red
*Z* = 4	0.35 × 0.15 × 0.1 mm

### Data collection

**Table:**

Bruker APEXII CCD diffractometer	5082 independent reflections
Radiation source: fine-focus sealed tube	4459 reflections with *I* > 2σ(*I*)
Graphite monochromator	*R*_int_ = 0.070
φ and ω scans	θ_max_ = 35.0°, θ_min_ = 3.5°
Absorption correction: multi-scan (SADABS; Bruker, 2014)	*h* = −17→19
*T*_min_ = 0.304, *T*_max_ = 0.417	*k* = −10→4
25308 measured reflections	*l* = −26→26

### Refinement

**Table:**

Refinement on *F*^2^	Primary atom site location: structure-invariant direct methods
Least-squares matrix: full	Hydrogen site location: mixed
*R*[*F*^2^ > 2σ(*F*^2^)] = 0.026	H atoms treated by a mixture of independent and constrained refinement
*wR*(*F*^2^) = 0.068	*w* = 1/[σ^2^(*F*_o_^2^) + (0.0362*P*)^2^] where *P* = (*F*_o_^2^ + 2*F*_c_^2^)/3
*S* = 1.03	(Δ/σ)_max_ < 0.001
5082 reflections	Δρ_max_ = 1.25 e Å^−^^3^
152 parameters	Δρ_min_ = −0.51 e Å^−^^3^
4 restraints	

### Special details

**Table:**

Geometry. All esds (except the esd in the dihedral angle between two l.s. planes) are estimated using the full covariance matrix. The cell esds are taken into account individually in the estimation of esds in distances, angles and torsion angles; correlations between esds in cell parameters are only used when they are defined by crystal symmetry. An approximate (isotropic) treatment of cell esds is used for estimating esds involving l.s. planes.

### Fractional atomic coordinates and isotropic or equivalent isotropic displacement parameters (Å^2^)

**Table:**

	*x*	*y*	*z*	*U*_iso_*/*U*_eq_	
Co1	0.49462 (2)	0.68360 (2)	0.24090 (2)	0.00839 (4)	
O1A	0.66165 (5)	0.60756 (10)	0.24434 (4)	0.01230 (11)	
O2A	0.67317 (6)	0.26293 (11)	0.27311 (4)	0.01275 (11)	
O1B	0.43294 (5)	0.41595 (10)	0.16212 (4)	0.01065 (11)	
O2B	0.34759 (6)	0.20179 (10)	0.05000 (4)	0.01437 (12)	
O1W	0.49903 (6)	0.45171 (10)	0.34088 (4)	0.01116 (11)	
H1W1	0.4417 (17)	0.463 (3)	0.3540 (16)	0.017*	
H1W2	0.5573 (18)	0.505 (3)	0.3843 (15)	0.017*	
O2W	0.29615 (6)	0.39794 (11)	0.37156 (4)	0.01612 (12)	
H2W1	0.2869 (13)	0.290 (2)	0.3380 (10)	0.024*	
H2W2	0.3027 (13)	0.344 (2)	0.4235 (8)	0.024*	
C1A	0.71817 (7)	0.43527 (13)	0.26448 (5)	0.01045 (14)	
C2A	0.85034 (8)	0.44468 (14)	0.28101 (6)	0.01530 (15)	
H2A	0.8621	0.5188	0.2314	0.018*	
C3A	0.90702 (9)	0.2299 (2)	0.28691 (9)	0.0292 (2)	
H3A1	0.9021	0.1576	0.3377	0.044*	
H3A2	0.9885	0.2457	0.2917	0.044*	
H3A3	0.8663	0.1508	0.2350	0.044*	
C4A	0.90875 (11)	0.5731 (2)	0.36327 (11)	0.0409 (4)	
H4A1	0.8738	0.7102	0.3563	0.061*	
H4A2	0.9918	0.5855	0.3726	0.061*	
H4A3	0.8976	0.5050	0.4130	0.061*	
C1B	0.36124 (7)	0.38036 (14)	0.08390 (5)	0.01115 (14)	
C2B	0.29432 (9)	0.56464 (14)	0.03069 (6)	0.01593 (16)	
H2B	0.2944	0.6787	0.0714	0.019*	
C3B	0.16552 (9)	0.50628 (19)	−0.02026 (7)	0.0238 (2)	
H3B1	0.1637	0.3970	−0.0616	0.036*	
H3B2	0.1245	0.6271	−0.0512	0.036*	
H3B3	0.1278	0.4583	0.0201	0.036*	
C4B	0.35856 (10)	0.64054 (17)	−0.03141 (6)	0.02213 (19)	
H4B1	0.4358	0.6907	0.0025	0.033*	
H4B2	0.3142	0.7519	−0.0676	0.033*	
H4B3	0.3660	0.5265	−0.0680	0.033*	

### Atomic displacement parameters (Å^2^)

**Table:**

	*U*^11^	*U*^22^	*U*^33^	*U*^12^	*U*^13^	*U*^23^
Co1	0.00921 (6)	0.00625 (7)	0.00890 (5)	0.00009 (3)	0.00197 (4)	−0.00013 (3)
O1A	0.0120 (3)	0.0092 (3)	0.0155 (3)	0.0012 (2)	0.0043 (2)	0.0020 (2)
O2A	0.0116 (3)	0.0097 (3)	0.0164 (3)	0.0002 (2)	0.0040 (2)	0.0016 (2)
O1B	0.0120 (3)	0.0083 (3)	0.0091 (2)	0.00017 (19)	0.00020 (19)	−0.00006 (17)
O2B	0.0182 (3)	0.0100 (3)	0.0114 (3)	0.0011 (2)	0.0003 (2)	−0.00154 (19)
O1W	0.0129 (3)	0.0097 (3)	0.0104 (2)	−0.0009 (2)	0.0032 (2)	−0.00094 (18)
O2W	0.0229 (3)	0.0121 (3)	0.0144 (3)	−0.0006 (2)	0.0076 (2)	−0.0006 (2)
C1A	0.0106 (3)	0.0103 (4)	0.0103 (3)	0.0002 (2)	0.0032 (2)	0.0001 (2)
C2A	0.0105 (3)	0.0139 (4)	0.0217 (4)	−0.0002 (3)	0.0057 (3)	0.0017 (3)
C3A	0.0138 (4)	0.0223 (6)	0.0489 (7)	0.0050 (4)	0.0071 (4)	−0.0045 (5)
C4A	0.0179 (5)	0.0483 (9)	0.0492 (8)	−0.0048 (5)	0.0013 (5)	−0.0285 (6)
C1B	0.0123 (3)	0.0107 (4)	0.0093 (3)	0.0010 (3)	0.0022 (2)	0.0000 (2)
C2B	0.0209 (4)	0.0117 (4)	0.0114 (3)	0.0047 (3)	0.0004 (3)	0.0006 (3)
C3B	0.0187 (4)	0.0263 (6)	0.0210 (4)	0.0078 (4)	−0.0005 (3)	0.0026 (3)
C4B	0.0326 (5)	0.0146 (4)	0.0179 (4)	−0.0008 (4)	0.0067 (4)	0.0039 (3)

### Geometric parameters (Å, º)

**Table:**

Co1—O1A	2.0449 (6)	C2A—C4A	1.5176 (16)
Co1—O2A^i^	2.0142 (6)	C3A—H3A1	0.9600
Co1—O1B	2.1100 (6)	C3A—H3A2	0.9600
Co1—O1B^i^	2.1198 (6)	C3A—H3A3	0.9600
Co1—O1W	2.1768 (6)	C4A—H4A1	0.9600
Co1—O1W^i^	2.1777 (6)	C4A—H4A2	0.9600
O1A—C1A	1.2755 (10)	C4A—H4A3	0.9600
O2A—C1A	1.2533 (10)	C1B—C2B	1.5179 (12)
O1B—C1B	1.2878 (9)	C2B—H2B	0.9800
O2B—C1B	1.2510 (11)	C2B—C3B	1.5340 (14)
O1W—H1W1	0.79 (2)	C2B—C4B	1.5329 (14)
O1W—H1W2	0.88 (2)	C3B—H3B1	0.9600
O2W—H2W1	0.861 (12)	C3B—H3B2	0.9600
O2W—H2W2	0.884 (11)	C3B—H3B3	0.9600
C1A—C2A	1.5191 (12)	C4B—H4B1	0.9600
C2A—H2A	0.9800	C4B—H4B2	0.9600
C2A—C3A	1.5187 (15)	C4B—H4B3	0.9600
			
O1A—Co1—O1B	88.13 (3)	C4A—C2A—C3A	111.50 (9)
O1A—Co1—O1B^i^	89.41 (3)	C2A—C3A—H3A1	109.5
O1A—Co1—O1W	92.18 (3)	C2A—C3A—H3A2	109.5
O1A—Co1—O1W^i^	88.29 (3)	C2A—C3A—H3A3	109.5
O2A^i^—Co1—O1A	175.30 (3)	H3A1—C3A—H3A2	109.5
O2A^i^—Co1—O1B	89.99 (3)	H3A1—C3A—H3A3	109.5
O2A^i^—Co1—O1B^i^	93.14 (3)	H3A2—C3A—H3A3	109.5
O2A^i^—Co1—O1W	91.70 (3)	C2A—C4A—H4A1	109.5
O2A^i^—Co1—O1W^i^	88.33 (3)	C2A—C4A—H4A2	109.5
O1B—Co1—O1B^i^	170.29 (2)	C2A—C4A—H4A3	109.5
O1B—Co1—O1W	79.22 (2)	H4A1—C4A—H4A2	109.5
O1B^i^—Co1—O1W	91.49 (2)	H4A1—C4A—H4A3	109.5
O1B—Co1—O1W^i^	110.31 (2)	H4A2—C4A—H4A3	109.5
O1B^i^—Co1—O1W^i^	78.99 (2)	O1B—C1B—C2B	118.13 (8)
O1W—Co1—O1W^i^	170.46 (2)	O2B—C1B—O1B	122.42 (8)
C1A—O1A—Co1	130.11 (6)	O2B—C1B—C2B	119.42 (7)
C1A—O2A—Co1^ii^	131.28 (6)	C1B—C2B—H2B	108.3
Co1—O1B—Co1^ii^	98.44 (2)	C1B—C2B—C3B	111.27 (8)
C1B—O1B—Co1	135.89 (6)	C1B—C2B—C4B	109.15 (8)
C1B—O1B—Co1^ii^	125.09 (6)	C3B—C2B—H2B	108.3
Co1—O1W—Co1^ii^	94.70 (2)	C4B—C2B—H2B	108.3
Co1—O1W—H1W1	109.1 (16)	C4B—C2B—C3B	111.30 (8)
Co1^ii^—O1W—H1W1	116.6 (14)	C2B—C3B—H3B1	109.5
Co1—O1W—H1W2	98.2 (15)	C2B—C3B—H3B2	109.5
Co1^ii^—O1W—H1W2	127.6 (14)	C2B—C3B—H3B3	109.5
H1W1—O1W—H1W2	106 (2)	H3B1—C3B—H3B2	109.5
H2W1—O2W—H2W2	103.8 (14)	H3B1—C3B—H3B3	109.5
O2A—C1A—O1A	124.94 (8)	H3B2—C3B—H3B3	109.5
O2A—C1A—C2A	118.59 (7)	C2B—C4B—H4B1	109.5
O1A—C1A—C2A	116.46 (7)	C2B—C4B—H4B2	109.5
C1A—C2A—H2A	107.6	C2B—C4B—H4B3	109.5
C3A—C2A—C1A	113.25 (8)	H4B1—C4B—H4B2	109.5
C3A—C2A—H2A	107.6	H4B1—C4B—H4B3	109.5
C4A—C2A—C1A	108.94 (8)	H4B2—C4B—H4B3	109.5
C4A—C2A—H2A	107.6		
			
Co1—O1A—C1A—C2A	−166.13 (6)	Co1^ii^—O1B—C1B—O2B	−16.84 (12)
Co1—O1A—C1A—O2A	12.38 (12)	Co1—O1B—C1B—C2B	−4.15 (12)
O1A—C1A—C2A—C3A	−168.74 (8)	Co1^ii^—O1B—C1B—C2B	165.09 (6)
O1A—C1A—C2A—C4A	66.57 (12)	O1B—C1B—C2B—C3B	−138.98 (8)
O2A—C1A—C2A—C3A	12.65 (12)	O1B—C1B—C2B—C4B	97.80 (9)
O2A—C1A—C2A—C4A	−112.03 (11)	O2B—C1B—C2B—C3B	42.89 (11)
Co1—O1B—C1B—O2B	173.93 (6)	O2B—C1B—C2B—C4B	−80.33 (10)

Symmetry codes: (i) −*x*+1, *y*+1/2, −*z*+1/2; (ii) −*x*+1, *y*−1/2, −*z*+1/2.

### Hydrogen-bond geometry (Å, º)

**Table:**

*D*—H···*A*	*D*—H	H···*A*	*D*···*A*	*D*—H···*A*
O1*W*—H1*W*1···O2*W*	0.79 (2)	1.91 (2)	2.6638 (10)	161 (2)
O1*W*—H1*W*2···O2*B*^i^	0.88 (2)	1.79 (2)	2.6206 (9)	158 (2)
O2*W*—H2*W*1···O1*A*^ii^	0.86 (1)	2.01 (1)	2.7967 (9)	151 (1)
O2*W*—H2*W*2···O2*B*^iii^	0.88 (1)	1.95 (1)	2.8087 (9)	163 (1)
C2*B*—H2*B*···O2*A*^i^	0.98	2.47	3.3094 (11)	144

Symmetry codes: (i) −*x*+1, *y*+1/2, −*z*+1/2; (ii) −*x*+1, *y*−1/2, −*z*+1/2; (iii) *x*, −*y*+1/2, *z*+1/2.
